# Editorial: Post-stroke complications: mechanisms, diagnosis, and therapies

**DOI:** 10.3389/fneur.2023.1292562

**Published:** 2023-09-27

**Authors:** Wenqiang Chen, Yinong Huang, Cheong-Meng Chong, Haiqing Zheng

**Affiliations:** ^1^Section of Integrative Physiology and Metabolism, Joslin Diabetes Center and Department of Medicine, Harvard Medical School, Boston, MA, United States; ^2^Department of Endocrinology, The First Affiliated Hospital of Sun Yat-sen University, Guangzhou, China; ^3^Center for Stem Cell Biology and Tissue Engineering, Key Laboratory for Stem Cells and Tissue Engineering, Ministry of Education, Sun Yat-sen University, Guangzhou, China; ^4^State Key Laboratory of Quality Research in Chinese Medicine, Institute of Chinese Medical Sciences, University of Macau, Macao, China; ^5^Third Affiliated Hospital of Sun Yat-sen University, Guangzhou, China

**Keywords:** stroke, complications, diagnosis, clinical trials, neuropathology

## Introduction

Stroke is a leading cause of disability and death that primarily affects arteries in the brain (1). When a stroke occurs, blood vessel blockages or narrowing restrict the supply of oxygen and nutrients to the brain, resulting in severe consequences including cell death and subsequent brain damage. Depending on the volume of ischemic lesion and the availability of immediate medical treatment following a stroke, the severity of post-stroke complications varies, spanning from temporary to permanent disabilities. These complications encompass pain, paralysis, language or swallowing difficulties, and sensory deficits, ultimately leading to psychiatric and cognitive impairments that profoundly impact patients' daily existence. While tremendous progress has been made in understanding the mechanisms underlying post-stroke complications in recent decades, the diagnosis and treatment of these complications are still in need of further development.

**Figure 1:**
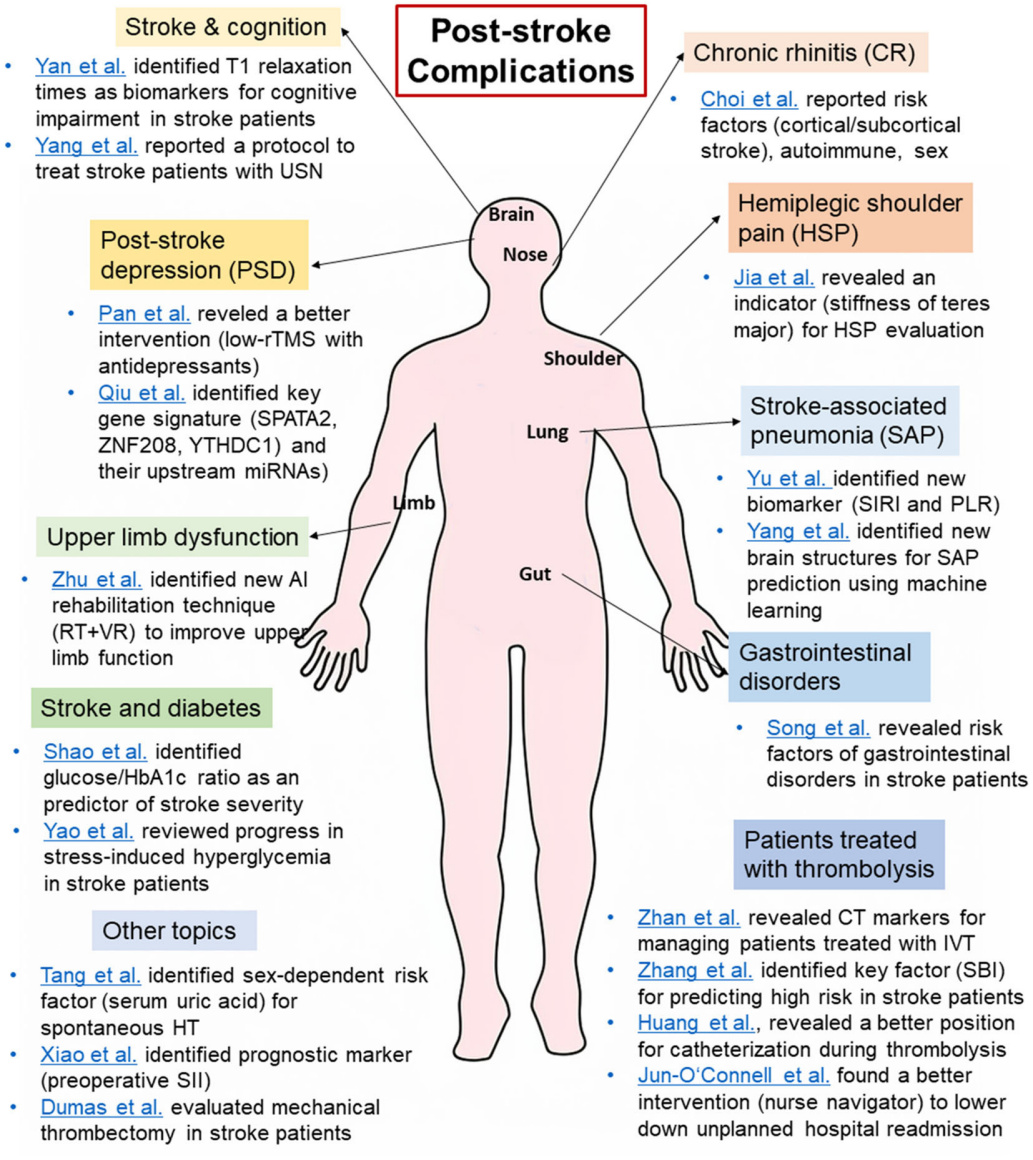
A summary of the main findings of the articles in the Research Topic “*Post-stroke complications: mechanisms, diagnosis, and therapies*”. CR, chronic rhinitis; HSP, hemiplegic shoulder pain, HT, hemorrhagic transformation; IVT, intravenous thrombolysis; PLR, platelet/lymphocyte ratio; PSD, post-stroke depression; rTMS, repetitive transcranial magnetic; SAP, stroke-associated pneumonia; SBI, silent brain infarction; SII, systemic immune-inflammation index; SIRI, systemic inflammation response index; USN, unilateral spatial neglect.

Within this Research Topic titled “*Post-stroke complications mechanisms, diagnosis, and therapies*,” we have presented novel research focused on different aspects of post-stroke complications. These studies advance our understanding of the pathogenesis and prognosis of post-stroke complications. In this editorial, our goal is to present a summary of the key findings in each published article. Besides the text discussion, a summary of the main findings of these articles is depicted in Figure 1.

## Post-stroke complications and inflammation

Systemic inflammation has been identified as an important factor that can impact both the early and long-term prognosis in stroke survivors (2). Within this Research Topic, we have three articles that delve into this subject.

First, Yu et al. perform a retrospective study that enrolls patients with spontaneous intracerebral hemorrhage (SICH). Through the analysis of data from these patients, the authors develop a predictive nomogram that incorporates factors such as systemic inflammation response index (SIRI) and platelet/lymphocyte ratio (PLR). They demonstrate the prognostic significance of these inflammatory biomarkers, outperforming conventional factors, in predicting stroke-associated pneumonia (SAP) following SICH. This study could help identify the risks of SAP, thus potentially improving patients' clinical outcomes and shorten the length of hospital stays.

Second, Xiao et al. conduct a retrospective study in patients with spontaneous basal ganglia intracerebral hemorrhage (ICH) who underwent surgical procedures. The authors discover that lower levels of preoperative systemic immune-inflammation index (SII) are linked to a reduced risk of prolonged mechanical ventilation (PMV) in these patients. Therefore, the study suggests that preoperative SII can serve as a prognostic marker for PMV.

Patients with stroke are reported to suffer from chronic rhinitis (CR), which results in symptoms such as rhinorrhea, nasal obstruction, and sneezing. Choi et al. conduct a retrospective study examining the association between CR and stroke in stroke patients. They find that, while clinical evaluation does not reveal a significant difference between CR and non-CR patients, patients with cortical and subcortical stroke are at a higher risk of developing CR. Furthermore, they identify autonomic symptoms and gender as risk factors for stroke patients to develop CR.

## Intervening and predicting post-stroke depression

Depression is commonly seen among individuals who have survived a stroke. According to the DSM-5, post-stroke depression (PSD) is characterized as a mood disorder with depressive features resulting from stroke (3). Our Research Topic has published three articles that reveal the key connection between PSD and stroke.

First, Pan et al. conduct a meta-analysis comprising 16 randomized controlled trials that enrolled 1,463 PSD patients. They find that combining low-frequency repetitive transcranial magnetic stimulation (low-frequency rTMS) with antidepressants leads to a significant reduction in depression scores, improved cognitive function, and lower levels of inflammatory factor when compared to antidepressant therapy alone. Furthermore, this analysis demonstrates that low-rTMS is generally considered safe with fewer adverse effects. Nonetheless, the authors recommend that future research should investigate the optimal intervention sites and frequencies for the treatment of PSD.

To investigate miRNA and mRNA biomarkers with predictive potential for PSD, Qiu et al. conduct a transcriptional analysis using data from two GEO databases that include patients diagnosed with both stroke and depression. Following the identification of differentially expressed miRNA and mRNA, the authors employ three machine-learning methods to uncover key signatures, including three genes (SPATA2, ZNF208, and YTHDC1) and their upstream miRNAs, forming a miRNA-mRNA network. This network could offer novel insights into the pathogenesis of PSD.

Another frequently occurring post-stroke complication, known as hemiplegic shoulder pain (HSP), has been reported to have a high prevalence of 80% among post-stroke patients even after recovery (4). HSP can evolve into a chronic condition and become associated with higher rates of depression, ultimately leading to poor quality of life (5). In order to examine the relationship between HSP and muscle stiffness, specifically focusing on internal rotation muscle stiffness, Jia et al. perform a prospective observational study. They enroll 20 stroke patients with HSP and 20 healthy controls and discover that increased stiffness of the teres major muscle is correlated with greater pain intensity and reduced shoulder mobility in patients with HSP. Thus, this study proposes that stiffness of internal rotation muscles can be used as an indicator for the evaluation and management pf HSP.

## Applying AI and machine learning tools to the treatment of post-stroke complications

Zhu et al. perform a network meta-analysis that includes randomized controlled trials. Through a comparison of the efficacy of six different artificial intelligence (AI)-based rehabilitation techniques aimed at enhancing upper limb function and daily living ability in stroke patients, the authors identify one technique that can effectively enhance both proximal and distal upper limb function. Thus, these findings not only indicate the potential advantages of AI-based interventions for stroke patients but also underscore the importance of taking patient characteristics into account in future research.

To predict SAP using a readily accessible approach like brain CT scans, Yang G. et al. introduce a registration method that aligns brain images from both CT and MRI scans. The authors use three machine learning models (logistic regression, support vector machine, random forest) based on these scans and extract image features pertaining to the distribution and lesion areas of ICH. The results reveal key brain structures that exhibit a strong correlation with SAP, as determined through feature extraction from the scanned images. This provides potential insights into predicting SAP and its relationship with brain lesions.

## Managing stroke patients receiving thrombolysis

Zhan et al. perform a retrospective study in which they analyze CT data from patients diagnosed with acute ischemic stroke (AIS) who underwent intravenous thrombolysis (IVT) treatment. The findings indicate that severe leukoaraiosis, severe brain atrophy, and a greater burden of cerebral small vessel disease (CSVD) are linked to an elevated risk of hemorrhagic transformation (HT) occurring within 24–36 h of IVT. Therefore, this study provides potential therapeutic strategies for preventing HT in stroke patients.

Zhang et al. perform a retrospective study on patients diagnosed with ischemic stroke who underwent IVT. Using clinical and neuroimaging data, the authors categorize the patients into groups with silent brain infarction (SBI) and those without (non-SBI group), revealing that patients with SBI have a reduced likelihood of achieving favorable functional outcomes at 3 months compared to non-SBI patients. Thus, the authors conclude that SBI is an independent factor that predicts a higher risk of unfavorable outcomes in AIS patients receiving IVT treatment.

Huang et al., perform a randomized, controlled clinical trial to assess the efficacy of different stereotactic minimally invasive catheter placement positions during urokinase thrombolysis for small- to medium-sized basal ganglia hemorrhages. The results show that catheterization along the long axis of the hematoma led to shorter catheterization times, reduced urokinase dosage, improved hematoma clearance, and fewer complications when compared to catheterization at the hematoma center. However, there are no significant differences in short-term patient outcomes as measured by NIHSS scores.

Unplanned readmission, which is defined as hospital admission for the same diagnosis within 30 days of discharge after the initial admission (6), has long been viewed as a challenge to healthcare performance and a potential risk to patients. It has been reported that unplanned readmission rates following a stroke are 20% within 30 days. To identify methods for reducing unplanned readmission, Jun-O'Connell et al. conduct a retrospective cohort study that enrolls 447 stroke patients who underwent thrombolysis. By utilizing a stroke nurse navigator team, comprising two professionally trained nurses with expertise in stroke care, the authors find a substantial reduction in unplanned readmission rates within the implementation period (i.e., 3 days following hospital discharge). This indicates that the nurse navigator intervention, which encompasses medication reviews and follow-up planning, contributes to improved outcomes in stroke patients treated with thrombolysis.

## Managing stroke patients with diabetes

Shao et al. examine the relationship between stress hyperglycemia and AIS through a retrospective study that enrolls patients with AIS. The study reveals that the primary functional outcome, the glucose-to-HbA1c ratio, is associated with more severe AIS, particularly in patients without diabetes. Thus, the authors conclude that the glucose-to-HbA1c ratio is an independent predictor of stroke severity in non-diabetic patients.

Yao et al. review stress-induced hyperglycemia (SIH) in AIS patients. SIH is characterized by elevated blood glucose levels during and after AIS, and it is linked to larger infarct sizes and poorer outcomes. Despite efforts to control it with insulin therapy, clinical outcomes continue to be unsatisfactory, pressing need for new therapeutic approaches. Furthermore, this review explores the definitions, mechanisms, and challenges in achieving effective glucose control in patients with AIS.

## Other topics

Yan et al. recruit patients with acute cerebral infarction (CI) and healthy controls and evaluate cognitive performance and early brain microstructural changes, revealing a significant correlation between T1 relaxation times in various brain regions and cognitive test scores. Specifically, CI patients exhibit a significantly reduced cerebral blood flow, reflecting dysfunction in brain microstructure. Furthermore, the authors identify T1 relaxation times in the right temporal and frontal lobes as potential biomarkers for cognitive impairment following acute cerebral infarction. Thus, this study suggests a link between brain microstructure and cognitive function through cerebral hemodynamics.

Yang Y.-x. et al. describe a study protocol for a prospective randomized controlled clinical trial. Their objective is to examine the efficacy of a combined therapeutic approach, namely, prism adaptation with eye movement training, for patients with unilateral spatial neglect (USN) following a stroke. After evaluations at baseline and post-intervention follow-ups, the authors seek to identify an innovative treatment strategy, thereby providing a new evidence-based treatment option for patients with USN.

Tang et al. perform a retrospective study to investigate the sex-dependent relationship between serum uric acid (UA) levels and the occurrence of spontaneous HT in ischemic stroke patients. They discover that elevated UA levels are independently linked to a higher risk of spontaneous HT in males but not in female patients. These findings indicate a sex-dependent association between UA and the occurrence of spontaneous HT in male patients who have suffered from ischemic stroke.

Song et al. investigate the potential link between stroke and common gastrointestinal disorders such as peptic ulcer disease (PUD) and gastroesophageal reflux disease (GERD). Using Mendelian randomization, the authors do not uncover any evidence suggesting that genetic predisposition to ischemic stroke directly influences the development of gastrointestinal disorders. However, the authors do identify an association between complications arising from intracerebral hemorrhage, specifically deep ICH, and an increased risk of developing PUD and GERD. Therefore, this study suggests a brain-gut axis connection that links stroke and common gastrointestinal disorders.

Dumas et al. perform a retrospective study to evaluate the impact of infarct laterality on functional clinical outcomes in AIS patients who had low ASPECT (0–5), the main quantitative score used by brain imaging studies including CT and MRI scans (7). The study enrolls patients with either intracranial internal carotid artery or middle cerebral artery occlusions with a low ASPECT score (0–5) and finds that clinical outcomes at day 90 post-stroke do not significantly differ based on the laterality of the stroke. This suggests that mechanical thrombectomy treatment is equally valuable regardless of stroke laterality in this patient group.

## Conclusion

In conclusion, although post-stroke complications still represent a significant challenge in stroke care and management, the articles published in this Research Topic represent a substantial contribution to enhancing our understanding of the mechanisms and diagnosis of, and therapies for, post-stroke complications. The newly identified diagnostic tools, including neuroimaging and biomarkers such as miRNA and mRNA, hold promise for the early detection of and intervention in post-stroke complications, thus potentially increasing the efficacy of treatment and improving the recovery journey for stroke survivors.

## Author contributions

WC: Conceptualization, Validation, Visualization, Writing—original draft, Writing—review and editing. YH: Validation, Writing—review and editing. C-MC: Writing—review and editing. HZ: Writing—review and editing.
